# Collaborating to co-produce strategies for delivering adolescent sexual and reproductive health interventions: processes and experiences from an implementation research project in Nigeria

**DOI:** 10.1093/heapol/czaa130

**Published:** 2020-11-06

**Authors:** Chinyere Ojiugo Mbachu, Ifunanya Clara Agu, Obinna Onwujekwe

**Affiliations:** 1 Department of Community Medicine, University of Nigeria; 2 Health Policy Research Group; 3 Department of Health Administration and Management, University of Nigeria

**Keywords:** Stakeholder, participatory research, partnership, implementation, strategy, adolescent, sexual health, reproductive health

## Abstract

Implementation science embraces collaboration between academic researchers and key stakeholders/implementers for the dual purpose of capacity building and context-adaptation. Co-production ensures that knowledge created with inputs from various groups of stakeholders is more reflective of local contexts. This paper highlights the experiences of academic researchers and non-academic implementers in collaborating to design implementation strategies for improving access to sexual and reproductive information and services for adolescents. Data were collected through primary and secondary sources. Detailed review of project documents such as minutes of research meetings, reports of workshops and outputs of group work activities enabled detailed description of the processes and steps of co-designing implementation strategies. Information on experiences and perspectives of benefits of the collaborative were collected through in-depth interviews of non-academic partners and focus group discussion with academic researchers. Narrative synthesis was done for information extracted through document review. Thematic analysis of qualitative interviews was done. The process of designing implementation strategies happened in three chronological steps of setting up the collaborative, selecting intervention areas and convening partners’ meetings to design strategies. Specific activities include stakeholder engagement, situation analysis, selection of intervention areas, designing the implementation strategies and pre-testing implementation tools. The process of analysing and selecting collaborators was iterative, and facilitated by having an ‘insider’ key informant. Working with key stakeholders enabled knowledge sharing and exchange among partners. Information sharing within the collaborative facilitated shifting of mindsets about adolescent sexual and reproductive health, and contextual adaptation of names and labels given to strategies. Co-producing implementation strategies with non-academic implementers enabled stakeholder ownership of implementation strategies and set the scene for their adoption in implementation settings. Some challenges of co-production of knowledge are that it is time consuming; involves several iterations that may influence coherence of strategies; involves multiple interests and priorities and poses a threat to fidelity.

**KEY MESSAGES**

- Stakeholder mapping is a critical first step in forming a collaborative which is diverse/representative and competent to design implementation strategies for delivering health interventions. This process could be facilitated by having an ‘insider’ key informant.
- Meaningful engagement of key stakeholders in co-designing strategies enables knowledge sharing and exchange among partners. Information sharing within a collaborative facilitates shifting of mindsets about adolescent sexual and reproductive health, and contextual adaptation of names and labels given to strategies.
- Co-designing implementation strategies with non-academic implementers improves ownership and sets the scene for adoption and acceptability of interventions in implementation research.

## Introduction

As health programmes and interventions are increasingly being developed and introduced into various settings to achieve health improvements, there is a need to understand how best they can be delivered across health systems and diverse settings. Implementation research (IR) examines practical ways in which innovations of proven efficacy and effectiveness can be transferred into practice, scaled-up and/or sustained in different settings (Measure Evaluation, 2012). It is largely recognized for its potential to maximize the beneficial impact of health interventions, and encompasses the identification of implementation problems, development and testing of practical solutions to these problems, and determination of how best to introduce and scale-up these solutions in various settings and health systems (Measure Evaluation, 2012; Peters *et al.*, 2013). To achieve improvements in programme implementation, research findings need to be integrated into practice. IR aims to integrate evidence-based interventions and research findings into health policy and practice by moving results from efficacy and effectiveness to scalability and sustainability in the real world of implementation. Successful integration of evidence-based interventions and research findings into practice relies on the support of policymakers, relevant implementers such as service providers and other key stakeholders. Therefore, IR is conducted within routine systems and real life settings and may involve one or more of the relevant systems’ stakeholders, including those involved in designing, managing and utilizing programmes, and whose contributions affect the planning, implementation, monitoring and outcomes of interventions.

IR embraces collaboration between academic researchers and key stakeholders/implementers such as policymakers, programme managers, health workers civil society organizations and non-government agencies (Goodyear-Smith *et al.*, 2015). This collaboration could be useful to academic researchers for identifying context-specific practice-based outcomes in implementation settings, and for gaining insight into how amenable the setting is for implementation (Rycroft-Malone *et al.*, 2015; Gagliardi and Dobrow, 2016). It could also be useful to key stakeholders/non-academics for upskilling or capacity building (Iedema and Carroll, 2011; Goodyear-Smith *et al.*, 2015). Overall, whether for academic or non-academic partners, this creates a sense of trust and empowerment, and breaks down conventional structures of research collaboration, allowing for multidirectional and interconnected approaches to knowledge creation (Djenontin and Meadow, 2018). Hence, collaboration among stakeholders in IR is integrated into the research process at the outset of problem identification, through to implementation of strategies and measurement of outcomes.

Similar to assertions about usability of research evidence for decision making, health improvements are constrained by implementation challenges arising from poor choice of strategies or unalignment to local contexts (Berman *et al.*, 2011; Mahlatji, 2013). Co-production of knowledge has the potential to contribute to health improvements by increasing integration of IR findings into practice. This is because knowledge created with inputs from various groups of stakeholders is likely to be more comprehensive and reflective of local contexts, diverse perspectives and actors’ interests (Barber *et al.*, 2011; Oliver *et al.*, 2015, 2019, Vindrola-Padros *et al.*, 2019). Co-production is defined as a ‘process where people intentionally try to collaborate on equal terms to develop more collective wisdom which can become a basis for making the quality of life better’ (Norma Ruth Arlene, 2017). The concept has been applied to various fields where scientific knowledge needs to be balanced with contextual information and experiences of local actors, on the basis that all forms of knowledge are valuable for making improvements (Djenontin and Meadow, 2018; Vindrola-Padros *et al.*, 2019). Its application to health systems research describes a culture of partnerships between academic researchers and practitioners or implementers to achieve common knowledge or shared understanding of a particular topic of interest (Hewison *et al.*, 2012; Marshall, 2014). This entails a prolonged period of engagement during which information is shared, different types of knowledge are negotiated and new knowledge is generated or updated.

Although evidence of processes, benefits, challenges and costs of co-productive research is well documented in literature (Rycroft-Malone *et al.*, 2016; Oliver *et al.*, 2019), not much has been reported about co-production of implementation strategies within IR projects. This paper contributes to an improved understanding of the added value of co-production in IR by highlighting the experiences of academic researchers and non-academic implementers in collaborating to design implementation strategies for improving access to sexual and reproductive health (SRH) information and services for adolescents. We set out by describing the processes and steps taken in co-designing the implementation strategies, including how the collaborative was formed, and how data were collected and analysed. This is followed by findings on partners’ experiences in the process, their perceptions of benefits and challenges of collaborating with other stakeholders, and lessons learned. These findings are discussed in light of existing literature on co-production for health research.

## Materials and methods

### Description of study area and project

The IR project is being implemented in Ebonyi state, south-east Nigeria by the Health Policy Research Group, University of Nigeria, Ebonyi state. Six communities were selected across the State based on teenage pregnancy rate and prioritization by State government for adolescent health interventions. Over 40% of the estimated population of 2.8million people in the State are under 15 years of age; and this population is expected to double by 2050 (USAID, 2017). Ebonyi state has been reported to have high rates of teenage pregnancy and maternal mortality (National Population Commission and ICF Macro, 2014).

The 5-year IR project adopts a community-embedded approach for addressing SRH needs of adolescents in rural and urban settings. The project commenced in March 2018 and specifically targets in-school and out-of-school adolescents aged 13–18 years in selected communities through interventions that will be delivered through schools, health sector, homes and community platforms. The project is being implemented in three phases:

- Phase 1—(1) Stakeholder engagement to secure buy-in and support of policymakers and key stakeholders; and (2) Situation analysis to determine the situation of adolescent SRH, and identify key interventions being implemented in the State.
- Phase 2—Design and implementation of strategies for delivering key interventions for improving adolescent SRH in the State.
- Phase 3—Evaluation of IR outcomes.

**Figure 1:**
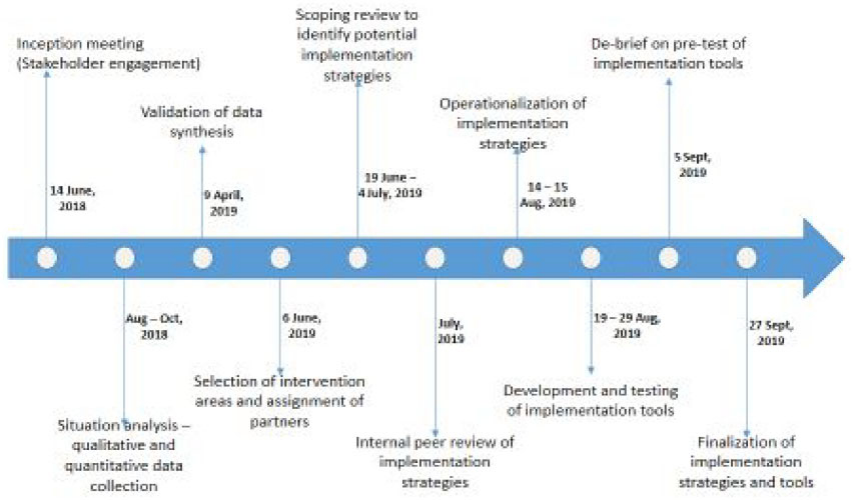
Chronological depiction of the process of co-producing implementation strategies

The process of co-production of implementation strategies by academic researchers and non-academic stakeholders began during the first phase of the IR project and it occurred in three chronological steps of setting up the collaborative, selecting intervention areas and convening partners’ meetings to design strategies (Figure 1). Detailed description of each step is presented subsequently.

Co-production was operationalized in this implementation context as the process of academic researchers and non-academic stakeholders working together as equal partners to develop and agree on the best approaches for delivering adolescent SRH interventions in the State.

### Step 1: Setting up a collaborative of researchers and non-academic stakeholders

At the inception of the IR project a 1-day stakeholder engagement meeting was organized with the objectives to, (1) introduce the project to key stakeholders in health in the State and get their buy-in; (2) obtain detailed information on policies, strategies and ongoing activities targeting adolescents’ SRH; (3) seek inputs on target communities, groups and potential interventions and (4) obtain commitment of government and non-government agencies to support implementation of the project and its knowledge translation in the State. This meeting, which was tagged a ‘*mini State Council on Health*’ by a Senior Special Adviser to the State Governor, attracted a diverse group of 62 key stakeholders comprising of high-level government officials, political office holders, traditional leaders, development partners, evidence-to-policy advocacy agencies, religious organizations and youth/adolescent peer groups. The meeting was structured into two plenary sessions and one parallel session during which participants were divided into three groups to deliberate on specific topics. Each group was given a reflection guide to aid their mapping of stakeholders working in adolescent health in the State. The guide was used to collect information on, (1) name of agency e.g. Ministry of Health (MoH); (2) key persons and positions in agency; (3) place of operation e.g. Abakaliki local government; (4) contributions to adolescent health or specific area of work and (5) key implementation challenges.

**Figure 2:**
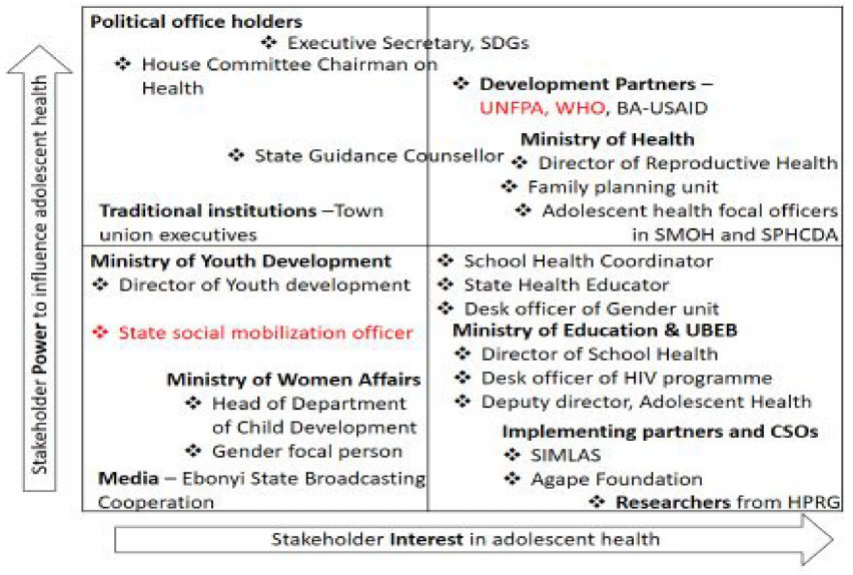
Mapping of Stakeholders' Influence and Interests in adolescent SRH in Ebonyi State

The lists of stakeholders from each group were synthesized and subsequently reviewed by a team of academic researchers and a key informant from the State, to ensure representation of all agencies working in adolescent health. Additional stakeholders were added from political office holders, traditional institutions and civil society groups to create a more comprehensive list. The revised list of stakeholders was used to select 24 people who were considered to be either significant contributors to adolescent health, key decision makers in the health sector or key influencers of adolescent health. Table 1 highlights the profile of all stakeholders in the revised list, and those who were selected to be part of the collaborative. An analysis of their levels of interest and alignment to adolescent SRH is shown in Figure 2. Stakeholders had varying levels of interest and influence on adolescent health. Although there was more clustering of implementers in the lower right quadrant (high interest, low power), a good balance of interest and influence was achieved by the number of stakeholders (implementers and policymakers) in the upper left and right quadrants (high power, low interest; and high power, high interest respectively) of the matrix.

**Table 1:** Profile of key stakeholders in adolescent health and those selected into the collaborative

Profile of all stakeholders identified during the workshop and subsequent review		Profile of stakeholders selected into the collaborative
**Political office holders & Legislators**		**Political office holders & Legislators**
1. Executive Secretary, Sustainable Development Goals		1. Executive Secretary, Sustainable Development Goals
2. House Committee Chairman on Health		2. House Committee Chairman on Health
**State Ministry of Health**		**State Ministry of Health**
3. Focal person for Adolescent health		3. Focal person for Adolescent health
4. Director of Reproductive Health		4. Director of Reproductive Health
5. Desk officer of Gender unit		5. Coordinator of Family planning unit
6. State social mobilization officer		6. Desk officer of Gender unit
7. Coordinator of Family planning unit		7. State Health Educator
8. State Health Educator		8. School Health Coordinator
9. School Health Coordinator		9. State social mobilization officer
**State Ministry of Women Affairs**		**State Ministry of Women Affairs**
10. Head of Department of Child Development		10. Head of Department of Child Development
11. Gender focal person		11. Gender focal person
**State Ministry of Education**		**State Ministry of Education**
12. Desk officer of HIV and AIDs programme		12. Desk officer of HIV and AIDs programme
13. Director of School Health		13. Director of School Health
**Universal Basic Education Board**		**Universal Basic Education Board**
14. Deputy director, Adolescent health unit		14. Deputy director, Adolescent health unit
**State Ministry of Youth, Development & Sports**		**State Ministry of Youth, Development & Sports**
15. Director of Youth development		15. Director of Youth development
**State Primary Healthcare Development Agency**		**State Primary Healthcare Development Agency**
16. Executive Secretary		16. Adolescent sexual health educator
17. Adolescent sexual health educator		**Development and implementing partners**
18. Adolescent health focal person, Afikpo south		17. UNFPA
19. Adolescent health focal person, Abakaliki		18. WHO
20. Adolescent health focal person, Ezza south		19. BA-USAID
**Health workers**		20. SIMLAS
Federal Teaching Hospital Abakaliki		**Civil society organizations**
**Development and implementing partners**		21. Agape foundation
22. UNFPA	23. Marie Stopes	22. Class Governor/Counsellor, HPHS
24. WHO	25. BA-USAID	**Others (Media & Traditional institutions)**
26. MCSP	27. SIMLAS	23. Ebonyi state broadcasting cooperation
28. AMURT		24. Town union executive, Ezza-Ama
**Civil society organizations**		Individuals/organizations who did not honour invitations to participate in the collaborative are highlighted in red
29. Agape foundation		
30. Class Governor/Counsellor, HPHS		
**Others (Media & Traditional institutions)**		
31. Ebonyi state broadcasting corporation		
32. Town union executive		
33. Traditional leaders		

### Step 2: Selection of key intervention areas and potential implementation strategies

Selection of key intervention areas was preceded by a situation analysis of adolescent SRH in Ebonyi state. Data were collected through quantitative survey of adolescents, aged 13–18 years, and qualitative interviews of policymakers, programme managers, health workers, community leaders, parents/guardians and adolescents. Findings from the situation analysis were synthesized and presented to selected stakeholders and study respondents in a workshop, for their reflections and endorsement. Participants identified three key interventions for improving adolescent SRH, (1) health information, (2) health services and (3) advocacy.

The team of academic researchers was divided into three groups of three or more researchers, including a senior researcher or a principal investigator in the project and each group was assigned an intervention area. Identification of potential implementation strategies for each intervention area was guided by an initial scoping review of relevant literature on implementation of adolescent SRH programmes in low- and middle-income countries. This was followed by internal peer review and series of meetings within and across groups to modify, define and harmonize potential strategies.

### Step 3: Convening partners meetings to co-produce implementation strategies

The next phase of the design of implementation strategies consisted of two major workshops and numerous minor meetings among partners in the collaborative. In the first major workshop, academic researchers and non-academic collaborators were introduced to the concept of implementation strategies including typologies, and how to specify or operationalize and justify the choice of a strategy for a given intervention. They were also introduced to the concept of IR outcomes and ways to measure these outcomes. Based on area of work and expertise, participants were divided into three intervention groups—information, health services and advocacy. Details of team composition are presented in Table 2. The lists of proposed strategies generated by academic researchers were presented and discussed in parallel groups, and modifications were made. Each group was asked to prioritize three strategies which will be used to deliver their respective intervention to adolescents, and to further describe/specify each strategy using Proctor *et al.*’s (2013) dimensions for adequate operationalization of implementation strategy.

**Table 2:** Composition of teams of collaborators in designing implementation strategies

Teams	Academic collaborators	Non-academic collaborators
**Advocacy**	1 senior researcher 1 mid-career researcher 1 early-career researcher	Executive Secretary, SDGs House Committee Chairman on Health—Legislator BA-USAID SIMLAS Agape foundation Town union executive, Ezza-Ama
Health information	1 senior researcher 1 mid-career researcher 1 early-career researcher	State Health Educator, SMOH School Health Coordinator, SMOH Desk officer of HIV & AIDs, SMOE Director of School Health, SMOE Deputy director, Adolescent health unit, UBEB Director of youth development Adolescent sexual health educator, SPHCDA Class Governor/Counsellor, HPHS Ebonyi state broadcasting cooperation
Health services	1 senior researcher 1 mid-career researcher 2 early-career researchers	Focal person for Adolescent health, SMOH Director of Reproductive Health & Nutrition, SMOH Coordinator of Family planning unit, SMOH Desk officer of Gender unit, SMOH
**Total**	10	19

Having operationalized implementation strategies and agreed on technical tools, each team of academic and non-academic collaborators began working on producing first drafts of implementation tools. Tasks were defined and shared within each team for sourcing documents such as existing manuals or protocols, reviewing documents and extracting relevant information, and synthesizing information into full drafts of respective tools. The advocacy tools—policy briefs—were developed using information from situation analysis adolescent SRH in Ebonyi state. Face-to-face and virtual meetings were held within and across teams to work on assigned tasks, peer review team outputs or provide updates on progress with designing tools.

The second major workshop was held after full drafts of implementation tools had undergone revision following peer review. The purpose of the meeting was to enable all members of a team to contribute in a penultimate review, refinement and restructuring of tools in preparation for pre-testing. The meeting lasted for 2 days and comprised parallel and plenary sessions. Revised versions of tools were presented in the last plenary and these were endorsed by participants for pre-testing.

The final phase of the design consisted of pre-testing and revision of document-type implementation tools. The tools were distributed to potential users and beneficiaries in another State and they were asked to read and report back whether, (1) the language is simple, (2) the information is clear and understandable, (3) the message in policy/advocacy brief is complete/comprehensive and convincing and (4) there are potentially controversial issues/topics that would require revision or careful attention during implementation. Feedback from the pre-test was shared during a de-brief meeting of academic researchers, and necessary revisions were made to the tools.

### Data collection

Primary and secondary sources of data were used. Primary data were collected through qualitative interviews of partners in the collaborative. The interviews were conducted by an experienced qualitative researcher who is a member of the research group but played a passive role of rotating through teams of collaborators to get a sense of the process and outcomes. Information on experiences and perspectives of benefits of the collaborative were collected through in-depth interviews of non-academic partners and focus group discussion with academic researchers. The decision to use different data collection methods was for the purpose of convenience for the interviewees and interviewer, and suitability of method to respondent types. We interviewed four randomly selected non-academic partners and eight academic researchers who attended the two face-to-face meetings. The non-academic partners were the State focal officers for reproductive health, adolescent health and school health and a representative of community-based organization working on adolescent health. We ensured that respondents were selected from each key intervention area, and only those who attended the two partners’ convenings were eligible. An interview guide was used to explore participants’ accounts of working with both academic and non-academic researchers in designing implementation strategies, in terms of how it influenced their views about adolescent SRH, as well as their choice of strategies. The guide also explored their perceptions of benefits and challenges of engaging with people on the ‘other side of the aisle’, and lessons learned in the process.

Secondary data were collected through detailed review of documents including minutes of research meetings, reports of workshops and outputs of group work activities during meetings/workshops. These reports were compiled by researchers who attended the workshops and meetings, and synthesized by a research assistant who is fully employed by the project. Each document was carefully read and relevant information was extracted to enable detailed description of the processes and steps of co-designing implementation strategies, including how the collaborative was formed, and the results and outcomes of each process.

### Data analysis

Narrative synthesis of information extracted from document review was done to describe in detail the process of co-designing implementation strategies and the strategies that were developed for implementing each key intervention area. Each implementation strategy is described in detail following Proctor *et al.*’s (2013) dimensions for adequate operationalization of implementation strategy.

The interviews were conducted in English language and audio recorded. Audio files were transcribed verbatim and transcripts were anonymized using pseudonyms. All transcripts were manually coded. A provisional list of codes/themes was generated are based on the research questions. An inductive approach (thematic analysis) of reviewing the data was then done to identify emerging constructs, patterns and experiences (Riessman, 2005). The broad steps in the thematic analysis were, (1) familiarization with the transcripts to identify recurrent/common themes—initial coding; (2) development of a coding scheme; (3) application of the coding scheme to all transcripts—descriptive coding; (4) sorting/grouping coded data to add a more detailed layer of meaning (interpretive coding). The final themes that were used in analysis are, (1) perceived effect of the collaborative on knowledge sharing; (2) perceived effect of information sharing on partners’ mindsets and views about adolescent SRH; (3) perceived effect of the collaborative on adoption and acceptability of strategies and (4) lessons learned from being a part of the collaborative.

## Results

In this section, we present implementation strategies that were prioritized by each team for delivering interventions to improve adolescent SRH. We also present partners’ perceptions of the effects of the collaborative on (1) knowledge sharing, (2) partners’ mindsets/views about adolescent SRH and (3) acceptability and adoption of implementation strategies.

### Implementation strategies for delivering interventions to improve adolescent SRH

The strategies that were prioritized by partners for delivering SRH services to adolescents include: (1) training of trainers (ToTs) on the provision of comprehensive SRH services to adolescents; (2) capacity building of primary health care (PHC) workers, community health workers (CHWs) and patent medicine vendors (PMVs) on the provision of adolescent-friendly SRH services and (3) supportive supervision of PHC workers, CHWs and PMVs (Table 3). These health service delivery strategies were selected because the baseline assessment uncovered a gap in the number of health workers who have been trained (and are skilled) to provide adolescent-friendly SRH services. Findings from the baseline assessment also showed that adolescents in the study communities rarely visited primary health centers to receive SRH services because of fear of lack of privacy and confidentiality, judgmental and negative attitudes of health workers and denial of care by health workers (Mbachu *et al.*, 2019). Rather, they preferred to go to PMVs and other informal providers who were more friendly and non-judgmental (Mbachu *et al.*, 2019). The decision to build a critical mass of trainers through ToT was to contribute to a sustainability plan beyond the life of the project. Capacity building of frontline health workers in the provision of adolescent-friendly health services, and deployment of CHWs have been shown to improve uptake of SRH services by adolescents and young people (Eke and Alabi-Isama, 2011; Brooks *et al.*, 2019).

**Table 3:** Strategies for delivering SRH information to adolescents

Name	Definition	Actors	Target	Actions	Temporality	Dose	IR outcomes & measurement
**Providing comprehensive SRH information to in-school adolescents**	This refers to activities aimed at providing teachers, guidance counsellors and peer educators with knowledge and skills to be able to provide SRH information to secondary school students; and creating a platform (through SHCs) for students to receive this SRH information from trained teachers and peers	Ministry of Education—MOE Universal Basic Education Board (UBEB) MoH State Guidance Counsellor Technical experts Teachers (secondary school) Students (peer educators) Local government education secretary	Adolescents in secondary schools	Capacity building workshop (ToTs—teachers, guidance counselors and peer educators) Establishment or reactivation of SHCs Provision of technical assistance to SHCs	**Early/initial phase—**Capacity building workshop and establishment of health clubs **Middle and last phase—**Technical assistance on possible ASRH activities	**Capacity building workshop and establishment of SHC—**once throughout the project life **Technical assistance—**once termly for one session (three times)	**Coverage:** Proportion of teachers, guidance counselors and peer educators who are trained; Number of SHC established; Proportion of adolescents who receive comprehensive SRH information through SHC. (Surveys and interviews) **Fidelity:** Adherence to guidelines for providing SRH information and setting up SHC. (Surveys and interviews) **Adoption:** Participants agree to establish SHCs and provide SRH information to students. (Feedback from training workshop)
**Community campaign on adolescent SRH**	This refers to all activities aimed at providing SRH information to out-of-school adolescents in their workplaces or skills acquisition centers; and through community fora such as youth days and football tournaments	MoH Ministry of Youth Development LGA social mobilization office Adolescent and youth networks and organizations Community leaders (including youth leaders) Media	Adolescents who are out-of-school (but does not exclude those in-school)	Identify and secure a venue or workplace or fora for the campaign Mobilize adolescents using appropriate channels Implement campaign activities—road walk; interactive sessions with adolescents	**Early/initial phase**—Identify and secure a venue or workplace or fora for the campaign **Middle phase**—mobilize adolescents and implement campaign activities	All actions shall happen once in corresponding implementation phases	**Coverage:** (a) Number of adolescents who participated in the campaign (b) Proportion of adolescents who received SRH information during the campaign (c) Proportion of adolescents who visited and/or received SRH services from trained providers after the campaign (Program report; Community survey)
**Parent–adolescent relationship education**	This refers to activities aimed at equipping parents with knowledge and skills they require to be able to initiate and provide comprehensive SRH information to their adolescents	Trained teachers and guidance counselors UBEB MoH—State health educator Technical experts Parents–Teachers Association (PTA) School principals	Parents and guardians of in-school and out-of-school adolescents	**Recruitment of parents** through PTA and village meetings **Training workshop for parents—**on adolescent SRH needs and communication with adolescents	All activities will happen in the **early/initial** phase of the project	All actions shall happen once in corresponding implementation phases	**Acceptability**—proportion of parents who honored the invitation for training and express a willingness to discuss SRH matters with their adolescents (Program report) **Coverage**—proportion of parents who are trained and who acquire communication skills in adolescent SRH (Community survey)

Regarding provision of SRH information to adolescents, the following strategies were prioritized: (1) training of secondary school teachers, guidance counsellors, peer educators and parents on provision of comprehensive SRH information to adolescents; (2) establishment of school health clubs (SHCs) on SRH and (3) community campaigns on adolescent SRH (Table 4). The first strategy was prioritized because the baseline assessment revealed several misconceptions among adolescents about pregnancy and sexually transmitted diseases (Mbachu *et al.*, 2020). Also majority of adolescents depended on ‘unreliable’ sources of information for their SRH knowledge, and parent–child communication of SRH matters rarely occurred (Mbachu *et al.*, 2020). Training of secondary school teachers would ensure that in-school adolescents have access to comprehensive, sequential SRH information. SHC has proven benefits of providing meaningful health education experience, facilitating school-community interaction and promotion of healthy lifestyle among students (Birch, 1986). It also fosters peer education which is an effective model for achieving behavioural change among adolescents (Eisenstein *et al.*, 2019; Biroudian *et al.*, 2020). It would also enrich the comprehensive information provided by teachers and guidance counsellors. Training parents and holding community campaigns on adolescent SRH were selected to ensure that out-of-school adolescents also get to receive information on SRH.

**Table 4:** Strategies for improving delivery of SRH services to adolescents

Name	Definition	Actors	Target	Actions	Temporality	Dose	IR outcomes & measurement
**Central level ToTs) on the provision of quality and comprehensive adolescent-friendly SRH services**	This refers to activities aimed at equipping senior and mid-level healthcare managers at the State level with knowledge and skills to be able to train frontline service providers to provide quality and comprehensive adolescent-friendly SRH services	MoH Primary Health Care Development Agency (PHCDA) Technical experts in adolescent health	Senior healthcare managers at State level Mid-level healthcare managers at State and LGA levels	Develop a training manual and training SOPs Capacity building workshop	**Early/initial phase**—Develop a training manual **Middle phase**—Capacity building workshop	All actions shall happen once in corresponding implementation phases	**Acceptability**—Number of invited trainers who attend the ToT workshop. Participants find the training and manuals useful for training frontline health workers **Adoption**—Participants express intention to train frontline health workers on provision of quality and comprehensive SRH services **Fidelity**—Adherence to training manual and SOP (Project reports)
**Capacity building of PHC workers, CHWs and PMVs on the provision of adolescent-friendly SRH services**	This refers to activities aimed at providing frontline health service providers with the knowledge and skills required to deliver quality and comprehensive adolescent-friendly SRH services	Trainers from TOT workshop: (1) senior healthcare managers at State level; (2) mid-level healthcare managers at State and LGA levels Technical experts in adolescent health	PHC workers CHWs PMVs	Develop separate training manuals and training SOPs for various categories of service providers (PHC workers, CHWs and PMVs) Capacity building workshops—parallel workshops for PHC workers, CHWs and PMVs	**Early/initial phase**—Develop a training manual **Middle phase**—Capacity building workshop	All actions shall happen once in corresponding implementation phases	**Coverage**—Number of service providers trained **Acceptability**—Participants find the trainings useful **Fidelity**—Adherence to training manuals and SOPs **Adoption**—Participants begin to provide adolescent-friendly SRH services in health facilities and communities (Project reports; IDIs and survey of service providers)
**Supportive supervision of PHC workers, CHWs and PMVs in the provision of adolescent-friendly SRH services**	This refers to activities aimed at reinforcing knowledge and skills required to deliver quality and comprehensive adolescent-friendly SRH services in the frontlines; as well as correcting errors that may be observed in this process	Trainers from TOT workshop: (1) senior healthcare managers at State level; (2) mid-level healthcare managers at State and LGA levels Technical experts in adolescent health	PHC workers CHWs PMVs	Develop supervisors checklist for various categories of service providers (PHC workers, CHWs and PMVs) Periodic (routine) supportive supervision visits to PHC workers, CHWs and PMVs	**Early/initial phase**—Develop supervision checklist **Middle and late phase**—periodic (routine) supportive supervision visits	Development of supervision checklist shall happen once in corresponding implementation phase. Periodic visits shall happen 1 month after the training and quarterly afterwards	**Adoption**—supervisors undertake routine supportive supervision of PHC workers, CHWs and PMVs **Coverage**—Number of frontline service providers visited and supervised as scheduled **Fidelity**—Adherence of supervisors to supervision checklist (IDIs and survey of service providers)

Advocacy to key decision makers and community leaders was deemed necessary to facilitate implementation of strategies for delivering SRH services and information to adolescents (previously presented). The first advocacy strategy is to lobby influential decision makers to institutionalize and facilitate the adoption of comprehensive sexuality education (CSE) in secondary schools. The second strategy consists of consultative forums with traditional rulers and community leaders to influence community support for access to CSE and health services for adolescents. The third advocacy strategy is a public panel discussion with adolescents, influencers and community leaders to discuss factors affecting access to CSE and health services for adolescents in Ebonyi state, and how participants can work together to address barriers to access for adolescents. These are fully operationalized in Table 5.

**Table 5:** Advocacy strategies for improving delivery of SRH services and information to adolescents

Name	Definition	Actors	Target	Actions	Temporality	Dose	IR outcomes & measurement
**Lobbying influential decision makers to institutionalize CSE in secondary schools**	Meeting decision makers who have strong influence in State health and education planning process: (1) to draw their attention to the need for CSE for adolescents, and (2) to make a clear request for institutionalization of sexuality education in all secondary schools	MOE UBEB MoH PHCDA Technical experts Political office holders NGO Ministry of Gender Affairs Adolescent and youth networks Media	Adolescents in secondary schools	Develop a briefing paper (policy brief) Visit each decision maker with a team of boundary partners Group advocacy meetings to foster sectoral collaborations	**Early/initial phase**—Develop a briefing paper **Middle phase**—lobbying meetings **Late phase**—lobbying meetings	Each influential decision maker shall be visited at least once	**Acceptability**—influential decision makers agree that CSE should be provided to adolescents and secondary school curriculum should be revised accordingly (feedback from lobbying meetings) **Adoption**—MOE and UBEB include/intend to revise secondary school curriculum to include CSE (feedback from lobbying meetings and IDIs)
**Consultative forums with traditional rulers and community leaders**	Inter-personal and group approaches (including dialogues) used to secure the commitment of traditional rulers and community leaders to bring about change in community attitude towards adolescents’ rights to comprehensive SRH information and services	MoH Ministry of Local Government and Chieftaincy Affairs Technical experts CSOs Adolescent and youth networks LGA health authority Ministry of Youth Development Media	Traditional Rulers Village heads Women leaders Religious leaders Community members Adolescents	**Develop an advocacy brief** Convene a **consultative forum** (meeting) with traditional rulers Individual visits to communities to meet with community leaders	**Early/initial phase**—development of advocacy brief **Middle phase**—consultative forum; follow -up meetings **Late phase**—follow-up meetings	All actions shall happen once in corresponding implementation phases	**Acceptability**—Traditional rulers agree that community support is needed in reviewing socio-cultural barriers to adolescent SRH (feedback from the consultative meeting) **Adoption**—Traditional rulers mobilize or intend to mobilize communities to review socio-cultural barriers to adolescent SRH (IDIs with community leaders)
**Public panel discussion with adolescents and experts**	Convene a meeting of adolescents and experts to exchange ideas and discuss on a topic of interest (‘access to SRH information and services’); explore factors that influence adolescent health and well-being; and discuss how participants can work together to improve access to comprehensive SRH information and services for adolescents.	MoH PHCDA Technical experts Political office holders NGOs/CSOs Development partners Adolescent and youth networks Media	Adolescents	**Recruitment of participants** comprising of discussants; moderator; audience (adolescents) **Planning the panel discussion**—goals; timing; presentation; questions for discussants; etc. **Convene the meeting Disseminate** resolutions through media and briefs	**Early/initial phase**—recruitment of participants; planning the discussion; convene panel discussion **Middle phase**—convene panel discussion; disseminate resolutions	All actions shall happen once in corresponding implementation phases	**Acceptability**—adolescents attend the panel discussion and agree that the topic is valid **Adoption**—participants (adolescents and experts) are willing to work together to create demand for quality CSE and health services among adolescents in the State

### Perceived effects of the collaborative on knowledge sharing

Prolonged and meaningful engagement of key stakeholders enabled knowledge sharing and exchange among partners. The face-to-face and virtual meetings enabled knowledge exchange between academic and non-academic partners in the collaborative. Academic researchers felt it was a rewarding and enriching experience because they had learned new things from partners on both aisles. Being that it was the first time of collaborating in research with non-academics for majority of the academic partners, they were pleasantly surprised to learn from the contextual knowledge of non-academics, and they found this very valuable in selecting implementation strategies and designing relevant tools. This knowledge exchange was sustained beyond the meetings as useful documents and materials were sourced by programme implementers in the State and shared with academic researchers in the collaborative.

*Well I could say that this session has been quite enriching and the experience is quite rewarding because we could see that from the wealth of their experience they (non-academic partners) were able to make significant contributions to the project* (Academic researcher, Female, R04).

*Engaging these stakeholders in getting implementation strategies is the right thing. Like today, they got some materials for us and I believe they will be very helpful in implementation for reaching communities because they will be able to communicate well with those in the community* (Academic researcher, Female R05).

*Our experts (partners) are wonderful in the sense that they are willing to give or share their experiences on adolescent sexual health. From the meeting we just concluded today, they had to tell us more things that we were not actually clear about when we were designing the strategies and it is now becoming clearer* (Academic researcher, Male R06).

Academic researchers considered engaging with non-academics as a win–win situation. They noted that interacting with programme experts was very beneficial because it drew their attention to issues and potential challenges to implementation which had not been well-thought-out.

*I also want to add that the benefit has actually been a dual thing because in a way, it helped to bring to bear some of the issues or challenges that our minds didn’t actually go to. For example the issue of ‘contraceptive’ in our project title* (Academic researcher, Female, R03).

Non-academic partners also considered it a learning experience. Although they had some experience in adolescent health programming, working with academic researchers enabled better conceptualization of adolescent SRH.

*It (working with researchers) has really influenced me because being in School health program, I have ideas of what adolescent SRH problem is all about. However, engaging with you people (researchers), has widened my knowledge. I am working with information team to help them fine tune their strategies and tools* (Non-academic partner, Female, IDI1).

### Perceived effects of information sharing on mindsets and views about adolescent SRH

Information sharing within the collaborative facilitated shifting of mindsets about adolescent SRH, and contextual adaptation of language. Partners’ on both sides of the aisle reported that the collaborative had influenced the way they think about adolescent sexuality, and this change in view was reflected in their selection and description of implementation strategies.

*I will say we (academic researchers) actually succeeded in changing their (non-academic partners) perception of adolescent sexuality. Most of them regarded adolescent reproductive health as same as contraceptives; now the orientation has changed and they are better informed and more welcoming of our ideas. And we are also benefitting from their experiences being that they have been in the field* (Academic researcher, Female R03).

*I joined this project during the design of implementation strategies, and it has opened my views to understanding the needs of adolescents, the risks they face, how they are not getting the right information from the right sources and what information and services they actually need* (Academic researcher, Male R06).

It was noted that in the early phases of the IR project (situation analysis), many partners in the collaborative found it uncomfortable discussing adolescent sexuality and access to contraceptives. Some partners felt it would be too suggestive to provide SRH information to adolescents, and were of the opinion that the discussion should be completely avoided. However, these views began to change as research evidence unearthed the situation of adolescent SRH in the State. Partners recognized the need to discuss the ‘elephant in the room’ and most of them became more accepting of the concept of adolescent sexuality and reproduction.

*Adolescent SRH especially contraception has been a subject area that people in our own context do not feel comfortable discussing. During stakeholders’ engagement meeting, some people were opposed to teaching adolescents about contraception, but after the baseline study and series of engagements in designing strategies I noticed that some who viewed discussing sexual matters with adolescents as ’forbidden’ started thinking differently. The engagement kind of made them to shift their mind and our stakeholders now feel that we must provide (SRH) information to adolescents, and contraceptive information for sexually active ones* (Academic researcher, Female R02).

Academic researchers also highlighted that through interacting and working with non-academic partners, some conventional terminologies that were used to specify/operationalize implementation strategies had to be modified to ensure context-sensitivity. For instance, academic researchers were advised that although the content of each strategy could be retained, their names/titles should be revised such that ‘contraceptive’ is replaced with ‘SRH’ anywhere it occurred alongside adolescents. It was explained that although communities accept that teenage pregnancy has become a public health problem that needs to be addressed, any intervention that is suggestive of sexual permissiveness for adolescents would be immediately shut down.

*The person in charge of adolescent reproductive health pointed out the issue of usage of some terms. For instance, she pointed out that although talking about contraception is very important we should have in mind that because we are going to be engaging with communities it is very important that we bring to the forefront the issue of abstinence so that we are not misunderstood. In fact she said there was a time a project like ours was to be started but religious bodies protested to government* (Academic researcher, Male R01).

### Perceived effect of the collaborative on adoption and acceptability of strategies

Involving non-academic implementers at the outset of the project was perceived to be a step in the right direction towards adoption of strategies for implementation of adolescent SRH interventions, and potential continuity beyond the research project.

*I just wanted to add that the fact that we engaged these experts is really the best practice. We know that sometimes, part of the reason why some projects are poorly implemented is when the people who should be involved, the communities or even the experts, are not involved. Because these people are now involved, it is something that can make a way even for sustainability to outlive the project itself* (Academic researcher, Male1).

Researchers felt that non-academic partners exhibited a sense of ownership and enthusiasm about the project because they were not just carried along, but were partners and participants in conceiving how interventions will be delivered in their communities and areas of practice. For instance, key decision makers in health service delivery co-designed strategies for improving access to SRH services for adolescents, while those in education and media sectors partnered to design strategies for delivering SRH information to adolescents.

*For me I think the important thing is this ownership and getting real context knowledge about so many things and then having access to all types of boundary partners and stakeholders. So I think it is very important and I think we have a good foundation right now* (Academic researcher, Male2).

*I think it has actually been worthwhile interacting or working with stakeholders in the state and the most interesting aspect is the enthusiasm partners have shown starting from the onset; the stakeholders’ engagement, planning stage, baseline study and now at the design stage. They have always shown their interest by coming out in numbers. Even when we are yet to schedule meetings, they will be the ones asking us, ‘when is it coming up?’ So the enthusiasm is there showing that it’s an area they are interested in carrying on, and are willing to partner with us* (Academic researcher, Female R03).

### Key lessons learned from co-producing implementation strategies

Detailed stakeholder mapping is required for careful selection of partners in a knowledge collaborative. Selection of non-academic partners was a non-linear process that required lots of iterations to ensure key partners were all represented in the collaborative. The process was guided by an initial stakeholder mapping (previously described). Some partners had to be included to ensure political support for implementation of strategies, as well as adoption and sustainability of strategies beyond project implementation cycle. Working with an ‘insider’ key informant in this process was perceived to contribute to a more diverse collaborative with adequate representation of non-academic partners. The insider key informant had spent considerable time in both academic/research and bureaucratic communities; and therefore had a good appreciation for balancing technical expertise with political representation in the collaborative.

The name and definition given to an implementation strategy should be sensitive and adaptable to implementation context because names matter. Naming or labelling and defining a strategy involves drawing upon same terms used by other researchers in the field. The name and definition given to a strategy provides a general sense of what that strategy may involve. Although not enough for full specification, naming and defining a strategy is important for distinguishing one from another (Proctor *et al.*, 2013). In our research, we set out with a title that had both ‘contraceptives’ and ‘adolescents’ as key terms because globally, contraception for adolescents is increasing being promoted to address unwanted teenage pregnancies and abortions. Collaborating with stakeholders in this research revealed that promoting contraceptives for adolescents is not as popular in the implementation context, and that naming a strategy with ‘contraceptive’ as a key term could constrain implementation by unacceptability of community leaders and non-participation of community members. Hence, in naming and defining strategies, IRs may have to change terminologies to ensure they are adaptable to implementation context, while not losing general sense of what the strategy involves.

Managing actors’ and their interests is required in a knowledge collaborative, and researchers develop such skills in the process. Navigating the process of selecting partners into a collaborative and co-designing implementation strategies with non-academics, enabled researchers gain experience in actor management. In designing implementation strategies, some programme managers and technocrats had preconceived ideas and notions of how best to deliver SRH services to adolescents and were determined to ensure that such strategies were included in the design. To minimize the influence of personal interests and biases in selecting implementation strategies, the research team adopted Proctor *et al.*’s (2013) dimensions for adequate operationalization of implementation strategy. In it, selection of strategies must be justified by first undertaking prospective assessment of factors that enable or prevent implementation in a given context, and providing clear justification for why selecting particular strategies would help in overcoming barriers and/or leveraging facilitators. The team also adopted Flottorp *et al.*’s (2013) recommendation that strategies should be selected because relevant theory, empirical evidence and/or pragmatic rationale suggest they may be appropriate to address specific challenges posed by implementation context. On these bases, partners were asked to reconsider any strategy that cannot be justified theoretically, empirically and/or pragmatically and in doing so, to set aside personal interests and biases.

Partners in a collaborative need to know what their roles are and when they would be required to make their contributions to the collaborative. At the very early stages of our project, more time was devoted to engaging with community leaders and political office holders whom we considered influential, rather than technocrats who had less political influence. The unintended outcome of doing this in our research project was that some technocrats felt side-lined and unappreciated compared with political office holders. Some programme managers felt that they were not being properly recognized and utilized for their technical expertise in adolescent health. One particular manager often complained that she was ‘*not being carried along*’ in the project. Although these views of being side-lined or unrecognized were reversed when programme managers became key partners in designing implementation strategies, it may have been averted by having clear communication plans at the outset of a research project to ensure all stakeholders were aware of when ‘their own time would come’. Communication plays a pivotal role in managing actors’ expectations by enhancing knowledge of their own and other people’s roles/contributions in IR process (Reynolds and Sariola, 2018).

## Discussion

Over a period of 15 months, academic and non-academic partners co-produced and pre-tested strategies for delivering adolescent health interventions. Our process aligns with the three-stage framework for co-production and prototyping of public health interventions which was used by Hawkins *et al.* (2017) to develop intervention content and delivery methods for school-based peer-led drug prevention interventions. Similar to the framework, our first stage consisted of stakeholder engagement and situation analysis (stakeholder consultation and evidence review), while the last two stages took the form of action-research consisting of face-to-face meetings, training workshops, email exchanges and feedback observations. The last two stages build on existing literature and theories on action and trans-disciplinary research (Stokols, 2006; Batalden *et al.*, 2011; Øvretveit *et al.*, 2011).

This collaboration resulted in the development of nine inter-dependent strategies that are reflective of the local context, representative of actors’ interest and diverse perspectives, and require multi-sector and multi-stakeholder inputs and actions. Non-academic partners in the collaborative ensured that strategies were operationalized in such a manner that they did not overtly contradict or conflict with societal norms and religious beliefs around adolescent sexuality (Oliver *et al.*, 2019; Vindrola-Padros *et al.*, 2019). Drawing from Flottorp *et al.*’s (2013) recommendations, academic partners on the other hand, contributed to ensuring that chosen strategies could be justified theoretically, empirically (using research evidence), and/or to a less extent, pragmatically (using experience). Hence, scientific justification of strategies was balanced with contextual information to produce strategies for delivering SRH interventions to adolescents, through a partnership that was adopted for knowledge exchange, negotiation and creation (Norma Ruth Arlene, 2017; Djenontin and Meadow, 2018; Vindrola-Padros *et al.*, 2019). The involvement of researchers, programme managers and intervention delivery staff shaped the content and structure of implementation strategies, and enabled ownership and willingness to adopt in implementation context among implementers.

Partners on both sides of the aisle benefitted from the knowledge sharing and exchange which occurred in the process of co-producing implementation strategies. While interacting with local actors, academic researchers gained better contextual understanding of how political, social, cultural and religious factors hinder access to SRH information and services for adolescents. For instance, although sexuality education is included in the national curriculum for secondary schools, it was not being implemented due to a lack of political interest and a reluctance of policymakers to debate or engage with strong conservative views of religious groups. This alerted the team of researchers to the need to strategize using targeted and sustained advocacy to key decision makers, community leaders and community influencers (including religious organizations). Non-academic partners also gained new knowledge of concepts in adolescent SRH, and a systematic approach of selecting strategies for delivering health interventions. This finding corroborates other reports that co-production benefits diverse stakeholders whilst influencing research, policy and practice interactions (Morris *et al.*, 2013; Beckett *et al.*, 2018).

A contextual learning from the implementation process was that information sharing within the collaborative facilitated shifting of mindsets about adolescent sexuality and reproductive health for both academic and non-academic partners. Partners with more traditional views about access to contraception for adolescents gradually changed their positions (and relaxed their views) as research evidence from the situation analysis was repeatedly shared to unearth the sore situation of adolescent SRH in the State. Those that were more open-minded in this regard also had to adapt some of their views to what society considers acceptable and appropriate. For instance, although the prevalence of teenage pregnancy is high in communities, interventions that suggest sexual permissiveness for adolescents (such as condom dispensaries in public places) would not be acceptable to community leaders. They would rather prefer that adolescents receive such services from health care workers. Consequently, all partners contributed equally in prioritizing and operationalizing the strategies.

Some of the key policy and health systems implications of this research in Nigeria is the use of the situational analysis to embark on an evidence-based development and deployment of the interventions. The evidence that was generated became the tool that was used to assuage the feelings of some stakeholders that were against implementing adolescent SRH interventions because of their religious, cultural and other beliefs. Hence, it is important that before such interventions are developed and implemented, there should be a robust situational analysis that involves collecting data from broad-based stakeholders that will include people that will be potentially against and for implementation of SRH strategies for adolescents.

In addition, the study recognized the importance of assembling, building and working in a broad-based multi-sectoral collaboration with diverse opinions to achieve some set objectives in advocacy and developing the interventions. This was in recognition of the importance of social determinants of adolescent SRH. Hence, stakeholders from key government ministries that were outside the Health sector, such as Ministries of Education and Information were made integral part of the team. This was in addition to the state SDG office and the State governor’s wife. This helped to give the project a stamp of State ownership for acceptability, integration into routine State-funded activities and sustainability at the end of the project. Hence, it is advised that in future, similar projects should adopt the approach of identifying important sectors and units to be involved in the exercise in their contexts through stakeholder mapping.

In the project, we attempted to achieve equal partnership by allocating partners to specific intervention groups (previously described), asking each group to scope for potential implementation strategies and to share these strategies for peer review. Equal partnership in a knowledge collaborative entails that every partner is given an opportunity to contribute ideas, information and experience without prejudice (Norma Ruth Arlene, 2017). It also presupposes that every partner’s contribution is valued and taken into consideration in making the final decisions (Djenontin and Meadow, 2018; Vindrola-Padros *et al.*, 2019), this implies ‘authentic collaboration as a context for action’, a situation where partners contribute to making a difference within and probably beyond the project (Rycroft-Malone et al., 2016; Cooke *et al.*, 2016). Although the initial scoping review was primarily undertaken by academic researchers, other partners in the group got a chance to review the list of strategies and include what was missing.

The co-production process was not without some challenges which have also been reported by similar works. It was a time consuming and engaging process with several iterations that made it difficult to track changes in the content of implementation strategies and ensure coherence of strategies across intervention areas (Cooke *et al.*, 2016; Hawkins, 2017). Second, the multidisciplinary and multi-sector nature of our research project entailed several interests and competing priorities between partners involved in the delivery of implementation strategies. Bridging these interest and priorities was an unanticipated task that researchers had to undertake, as previously discussed (Stokols, 2006; Hawkins, 2017). Another potential weakness which we anticipate is a deviation from guidelines and standard operating procedures for delivery of implementation strategies after co-production has ended and implementation tools are finalized. This poses a threat to fidelity in formal delivery if implementers are unable to adhere to intervention delivery guidance (Hawkins, 2017). Co-production of implementation strategies in this research followed a rigorous and systematic process, involving a team of experienced implementers and knowledgeable researchers. Implementation strategies were operationalized and justified using theoretical, empirical and pragmatic knowledge. However, caution should be exercised in adopting the strategies presented in this paper because they are still being rolled-out and implementation outcomes are being monitored, which will be reported in a subsequent paper.

## Conclusion

Co-production of knowledge with academic researchers, programme managers and other intervention delivery staff enabled knowledge sharing and exchange which was useful for creating robust context-adaptive implementation strategies for delivering adolescent SRH interventions. The iterative process consisted of stakeholder engagement, situation analysis, selection of intervention areas, co-producing implementation strategies and pre-testing implementation tools. Working with an ‘insider’ key informant facilitated formation of the knowledge collaborative. Engagement of key stakeholders, and information sharing facilitated shifting of mindsets, and enabled contextual adaptation of names and labels given to strategies. Co-producing implementation strategies with non-academic implementers improves ownership and sets the scene for adoption of strategies and acceptability of interventions in IR.

## Funding

The research leading to results included in this manuscript has received funding from IDRC MENA+WA implementation research project on maternal and child health (IDRC grant number: 108677). The views presented in this manuscript do not necessarily represent the funders’ views and belong solely to the authors. The authors appreciate the invaluable contributions of all partners in the collaborative. This article is part of the supplement ‘nnovations in Implementation Research in Low- and Middle-Income Countries’, a collaboration of the Alliance for Health Policy and Systems Research and Health Policy and Planning. The supplement and this article were produced with financial support from the Alliance for Health Policy and Systems Research. The Alliance is able to conduct its work thanks to the commitment and support from a variety of funders. These include our long-term core contributors from national governments and international institutions, as well as designated funding for specific projects within our current priorities. For the full list of Alliance donors, please visit: https://www.who.int/alliance-hpsr/partners/en/.

*Conflict of interest statement*. None declared.

*Ethical approval*. Ethical approval was obtained from the Ethics Committee of Ebonyi State Ministry of Health, and the Health Research Ethics Committee of University of Nigeria Teaching Hospital.
